# Integrated Flight Path Planning System and Flight Control System for Unmanned Helicopters

**DOI:** 10.3390/s110807502

**Published:** 2011-07-28

**Authors:** Shau Shiun Jan, Yu Hsiang Lin

**Affiliations:** Department of Aeronautics and Astronautics, National Cheng Kung University, Tainan 70101, Taiwan; E-Mail: yu_hsiang_lin@mail2000.com.tw

**Keywords:** unmanned helicopter, flight path planning system, flight control system, digital elevation model

## Abstract

This paper focuses on the design of an integrated navigation and guidance system for unmanned helicopters. The integrated navigation system comprises two systems: the Flight Path Planning System (FPPS) and the Flight Control System (FCS). The FPPS finds the shortest flight path by the A-Star (A*) algorithm in an adaptive manner for different flight conditions, and the FPPS can add a forbidden zone to stop the unmanned helicopter from crossing over into dangerous areas. In this paper, the FPPS computation time is reduced by the multi-resolution scheme, and the flight path quality is improved by the path smoothing methods. Meanwhile, the FCS includes the fuzzy inference systems (FISs) based on the fuzzy logic. By using expert knowledge and experience to train the FIS, the controller can operate the unmanned helicopter without dynamic models. The integrated system of the FPPS and the FCS is aimed at providing navigation and guidance to the mission destination and it is implemented by coupling the flight simulation software, X-Plane, and the computing software, MATLAB. Simulations are performed and shown in real time three-dimensional animations. Finally, the integrated system is demonstrated to work successfully in controlling the unmanned helicopter to operate in various terrains of a digital elevation model (DEM).

## 1. Introduction

Over the past few decades, Unmanned Aerial Vehicles (UAVs) [1] have received attention in many countries. Because there is no need for onboard pilots to operate the vehicle, UAVs have obvious advantages in difficult missions and dangerous environments. Furthermore, UAVs are deeply involved in the development of many important technical innovations, e.g., autopilots, path planning algorithms, and wireless communication.

In general, an unmanned helicopter is controlled by a remote pilot or it flies autonomously according to the pre-programmed flight plans created by sophisticated dynamic automation systems. Both methods have their advantages and disadvantages. Extensive human efforts are needed for the first method, because one pilot can only control one unmanned helicopter from the remote location at one time. The pilot may not be able to determine the least-cost path and guarantee the safety of the flight path at the same time. For the second method, the flight path is pre-programmed, and it cannot be changed during missions. If some unknown obstacles suddenly occur along the pre-programmed path, the unmanned helicopter would not be able to respond to the obstacles.

The core technology that combines the two unmanned helicopter control methods and thus enables unmanned flight is the navigation and guidance system [2]. However, two systems, the Flight Path Planning System (FPPS) and the Flight Control System (FCS), are necessary in order to complete the navigation and guidance system for an unmanned helicopter. The FPPS surveys the terrain and then generates a proper flight path for the FCS. The FCS maintains the unmanned helicopter in a stable state and completes the missions.

This paper presents a successful implementation of automatic, real-time path-planning algorithm coupled with a simple fuzzy flight controller on an unmanned helicopter which is commonly accepted to be more difficult than fixed-wing aircraft. The successful implementation of the FPPS and FCS for an unmanned helicopter is the main contribution of this work. For instance, before the expert experience is implemented on an actual unmanned helicopter, it can be tested by the proposed system. The high modularity of the proposed system makes it easy to put the expert experience into the FISs. It helps the development of the unmanned helicopter system using fuzzy logic and the expert experience.

### 1.1. Objective

The major objective of this paper is to design a navigation and guidance system for unmanned helicopters. One important part of the navigation and guidance systems proposed in this work is the FPPS. In this paper, the FPPS is designed by using the A-Star (A*) algorithm [3] which is a best-first graph search algorithm to plan the flight path, and the system is able to reduce the computation load and improve the efficiency [4]. Moreover, the FPPS developed in this work is capable of generating a flight path that excludes any predefined forbidden zone. If the onboard sensors detect a new obstacle in the Digital Elevation Model (DEM) during flight, the FPPS is able to alter the flight path instantaneously.

After the flight path is planned, it is forwarded to the FCS. In this paper, the FCS is designed based on a fuzzy logic control system, the Fuzzy Inference System (FIS) [5]. Instead of the differential equations and the dynamic model, the FIS determines the control solution by prior experience, expertise, and fuzzy logic. The FCS could guide the unmanned helicopter to follow the planned flight path and complete the designated task. Accordingly, an integrated test bed for the FPPS and the FCS is developed in this paper.

### 1.2. Research Survey

In previous research, several algorithms such as the potential fields’ algorithms [6,7], the graph search algorithm [8] and the evolutionary algorithm [9] were developed and applied to the FPPS. For the path finding problems, the graph search algorithms such as the Dijkstra algorithm [10] is used to find the shortest paths from a single grid to all other grids in the graph. The A-Star (A*) algorithm is similar to the Dijkstra algorithm but it uses a heuristic estimation to guide itself rapidly to the destination [11]. Evolutionary algorithms use Artificial Intelligence (AI) methods, such as the genetic algorithm and the neural network [5], to derive the heuristic function to help solve path finding problems. The Dynamic Programming (DP), which relies on the principle of optimization, is another known method of finding the shortest flight path [12]. The A-Star algorithm is closely related to DP. Furthermore, DP has the advantage of reduced computation time and it exhibits the properties of overlapping problems and optimal substructures [13]. Therefore, DP has been widely applied in robot motion and flight path planning.

A control system manages, commands, directs or regulates the behavior of other devices or systems. There are two common classes of the control systems: the logic controller and the feedback controller [5]. The fuzzy logic controller is one of the logic controllers, and it is an attempt at rather easy design and to control continuously varying systems [14,15]. Basically, a measurement in a fuzzy logic controller can be partly true. That is, if “Yes” is given the value “1” and “No” the value “0”, then a fuzzy measurement can have a value between 0 and 1 [5]. A database of expert knowledge and experience is constructed, and the fuzzy logic controller infers the value according to the database. The controller determines the proper outcome and finally the fuzzy logic controller converts the fuzzy set to a real world output to control the real machinery.

In 2006, the Remotely Piloted Vehicle and Microsatellite Research Laboratory (RMRL) at the Institute of Aeronautics and Astronautics (IAA) of National Cheng Kung University (NCKU) built a path planning system using a fast graph-search algorithm to find a feasible flight path for an UAV to traverse multiple targets [4]. Based on the real flight conditions and the limitations of the UAV’s performance, the system constructed a virtual terrain as a search space above the real terrain. The idea of a virtual terrain in [4] can eliminate a significant amount of search space from the 3-D plot to the 2-D to reduce computation time. In recent years, the RMRL has also been concentrating on the development of payload systems. At the same time, the Communication and Navigation Systems Laboratory (CNSL) in IAA of NCKU focuses on the integration of UAV onboard sensors to provide better performance. Currently, the CNSL and the RMRL cooperate to develop the navigation and guidance system for unmanned helicopter and the core of the research is to hover in the space to survey or track objects of interest.

### 1.3. Paper Organization

An integrated system of the FPPS and the FCS for the unmanned helicopter is developed in this paper, which is organized as follows. In Section 2, the virtual flight map is established above the DEM, and then the FPPS schemes the flight path according to the created virtual flight map. There are three methods used in Section 3 to improve the FPPS. First, the forbidden zone is designed to prevent the flight path from passing through dangerous zones or bad weather areas. Second, the Multi-Resolution Scheme is used to reduce the computation time. Finally, two Path Smoothing Methods are applied to improve the flight route quality [4]. The control, guidance and the FCS of the unmanned helicopter are detailed in Section 4. In Section 5, the adaptability and robustness of the integrated system that combines the FPPS and the FCS is demonstrated. The integrated system is simulated by integrating the computing software, MATLAB [16] and the flight simulation software, X-Plane [17], and the unmanned helicopter response is shown by three-dimensional animations in real time. Finally, Section 6 summarizes the work presented in this paper.

## 2. Flight Path Planning System

The function of the flight path planning system (FPPS) is to determine, while meeting safety requirements, the least-cost flight path. The flight path is actually a subset of three dimensional grids selected from the virtual flight map that the unmanned helicopter flies from one to the next until it arrives at the destination. The virtual flight map is a set of grids above the terrain with their horizontal projections distributed uniformly. Because the FPPS is designed for unmanned helicopters, the planned flight path should always be higher than some minimum acceptable altitude. That is, the altitude of each grid in the virtual flight map should always be higher than some minimum acceptable altitude. It is thus reasonable to say that safety parameters are incorporated to specify the minimum acceptable altitude in the virtual flight map construction. The path planning algorithm then determines the proper flight path from the virtual flight map.

### 2.1. Virtual Flight Map

**Figure 1.**
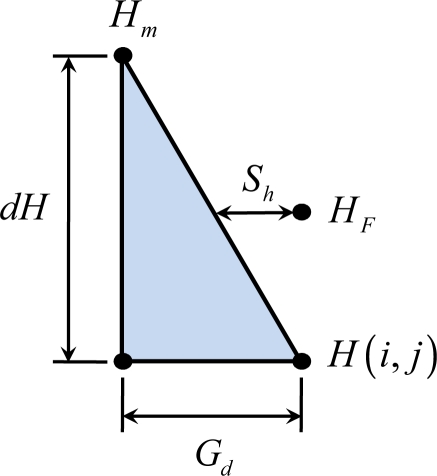
The example diagram of Equation (1).

Because the FPPS runs on the unmanned helicopter’s onboard computer, it would be time consuming if the FPPS explores the map and checks the safety of the grids at the same time. In order to simplify the safety check process, the unmanned helicopter flight speed is assumed to be constant in static environment [15]. Grids are considered to be scattered uniformly over the local sea level plane at the operation area. On each grid the terrain elevation, the horizontal safety distance, and the vertical safety distance are specified. The FPPS’s job now is to determine the virtual flight map altitude on that grid. To do this the cruise altitude known from the mission requirements is compared with the vertical safety distance on that grid and with the terrain elevations on eight neighboring grids. If the cruise altitude is higher than the vertical safety distance and the terrain elevations, the cruise map altitude is considered as the virtual flight map altitude. On the contrary, if the cruise altitude is lower than one of the terrain elevation, the virtual flight map altitude is determined by:

(1) $$H_{F}=H(i,j)+\frac{S_{h}\times dH}{G_{d}}$$

where *H_F_* is the virtual flight map altitude above grid *H*(*i*, *j*), *i* and *j* are the lateral and the longitudinal distances of the local sea level plane, respectively, *Gd* is the grid size, *S_h_* is the horizontal safety distance, and *dH* is the difference between the selected grid and the surrounding grids. An example of Equation (1) is shown in Figure 1.

**Figure 2.**
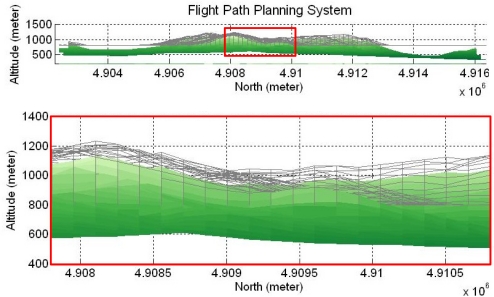
The virtual flight map is created above the real terrain.

Finally, the virtual flight map is constructed by examining the safety of all grids. Figure 2 is the virtual flight map meshed above the real terrain, and the bottom sub-figure is the zoom-in area of the red rectangle of the upper one. The FPPS works out the safe flight path using the virtual flight map, and the grid size of the virtual flight path is 150-by-150 meters. The real terrain used in this paper is the built-in terrain in the MATLAB Mapping Toolbox.

### 2.2. Path Planning Algorithm

After the virtual flight map is created, the FPPS continues with the A-Star algorithm. Similar to other search algorithms [8], the A-Star algorithm requires a starting point, a destination, and some flight parameters such as the fuel consumption, the rate of climb, and so on. The FPPS can thus arrange a proper flight path for various unmanned helicopters according to the different flight parameter settings.

In the A-Star algorithm, a cost function assigns a cost value to each grid. Equation (2) shows the cost function:

(2) F(x)=G(x)+H(x)

As shown in Equation (2), the cost function is the sum of a movement cost function *G*(*x*) and a heuristic function *H*(*x*). The movement cost function is chosen such that the value at a grid reflects the cost of going from the starting point to that grid under certain cost criteria. For example, the cost criteria used in this work are 10 units for horizontal and vertical movements and 14 units for diagonal movement. This way, the cost function is the actual shortest distance from the starting point to the grid. However, knowing the cost from the starting point to a grid is not sufficient for path planning. An estimate of the movement cost from that grid to the destination, represented by the heuristic function value at the grid, should also be considered. Different heuristic functions are possible for path planning, and the one used in this work follows:

(3) $$H(x)=h_{V}\times \sqrt{a^{2}+b^{2}}$$

where *h*_V_ is the heuristic parameter equal to the mission ratio, and *a* and *b* are the lateral distance and the longitudinal distance from the current grid to the destination, respectively. Straight route tends to be planned for high *h*_V_ values. It is emphasized that different cost and heuristic function can be used in the path planning algorithm regarding to different considerations.

The A-Star algorithm starts from the starting point. The cost value of the eight neighboring grids around the starting point is calculated, except the obstacle grid. The *G*(*x*) value and the *F*(*x*) value of the neighboring grids are stored in an open table. For each grid in the open table, the starting point is also recorded in the open table and is called the parent grid. The starting grid, together with the values it has in the open table, is now moved to a closed table. The above process repeats with the least *F*(*x*) value neighboring grid acting as a new starting grid. It is possible that the some of the new neighboring grids are already in the open table. This means that, aside from the current path, there is another path from the starting point to those neighboring grids already in the open table. If one of these grids happens to be the least *F*(*x*) value grid, the one with smaller cost value must be taken. That is, if the cost of the current path is less, the current *G*(*x*) value, *F*(*x*) value, and the new starting point are put in the closed table. On the other hand, the old *G*(*x*) value, *F*(*x*) value, and the old parent grid are put in the closed table. The whole process repeats until the helicopter arrives at the destination.

**Figure 3.**
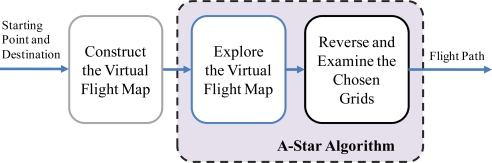
The block diagram of the developed FPPS.

At this stage, the conventional A-Star algorithm works out the flight path by navigating backwards the close to the starting point. It is noted that the least *F*(*x*) does not necessarily go back to the starting point. When the path with the least *F*(*x*) cannot go back to the starting point, it is eliminated. Then, the next least cost path is considered. Finally the flight path is planned after the backward determination process. The architecture of the developed FPPS is summarized in Figure 3.

**Figure 4.**
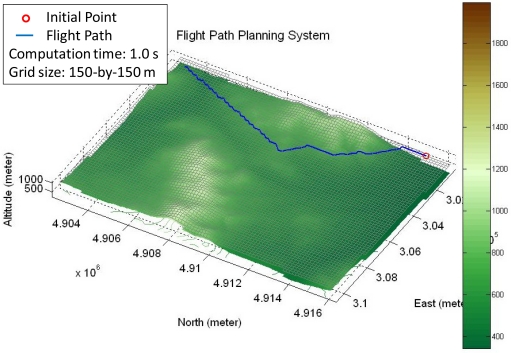
The resulting flight path by the developed FPPS.

Figure 4 shows the resulting flight path proposed by the developed FPPS, and the same virtual flight map shown in Figure 2. The blue line is the flight path and the circle is the starting point. The computation time of this example path is less than 1 second. Based on the mission ratio, the FPPS is able to plan different flight paths.

**Figure 5.**
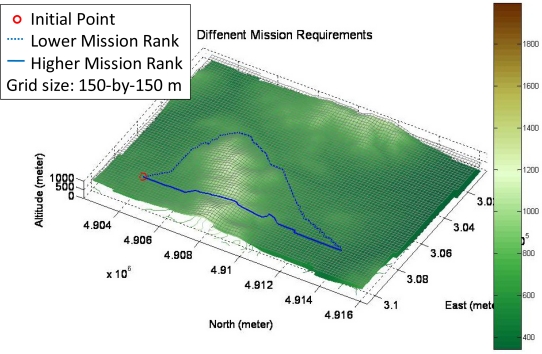
Two flight paths are planned according to the different mission requirements.

Figure 5 shows two planned flight paths according to different mission requirements. The dashed blue line indicates the path for the lower mission ratio, and the solid blue line shows the path for the higher mission ratio for the same starting point and destination. The higher mission ratio it is, the shorter path the FPPS plans. In that situation, the fuel consumption rate is high as well. Conversely, the lower rank corresponds to the least cost and smooth path, but more time is needed to achieve the destination. In this paper, the unmanned helicopter is assumed to be controlled by a remote pilot only for the takeoff and landing phases, and the FPPS is used to calculate the flight path for the remaining flight phases. Additionally, the developed FPPS is capable of planning the flight paths for different unmanned helicopters if the flight parameters of the other unmanned helicopters are set accordingly.

## 3. System Improvements

In this section three methods are presented to further improve the flight safety and reduce the computation time for the FPPS. First, the forbidden zone is used to improve flight safety [4]. Second, Multi-Resolution Scheme is applied to reduce the computation time of the FPPS [4]. Finally, the Path Smoothing Method is used to improve the quality of the planned flight path.

### 3.1. Forbidden Zone

**Figure 6.**
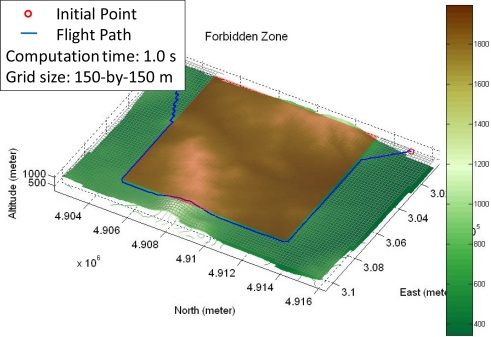
The red area is the forbidden zone on the virtual flight map.

Before the FPPS plans the flight path, the Digital Elevation Model (DEM) must be acquired to create the virtual flight map. The newest DEM, however, may not be available. Unexpected obstacles and changes of the real terrain may not be shown on the virtual flight map. The weather of the local area is another important factor. The unmanned helicopter may be in danger when the weather conditions are severe. Therefore, the forbidden zone is a necessary design feature to prevent the unmanned helicopter from entering a dangerous area. The forbidden zone is a square matrix on the virtual flight map [4], and the system eliminates the grids in the forbidden zone from the virtual flight map before planning the flight path. Figure 6 shows a forbidden zone on the virtual flight map, where the red area is the forbidden zone (from 300,000 to 309,000 meters East and from 4,905,000 to 4,913,000 meters North.) The flight path allows the unmanned helicopter to stay out the forbidden zone and arrive at the destination, and the computation time of the added process is no more than 1 second for this example.

### 3.2. Multi-Resolution Scheme

The computation time of the FPPS process depends mainly on the virtual flight map grid size [4]. Although the virtual flight map constructed by smaller grids can reflect the terrain details better, it compromises the FPPS computation time. Therefore, the Multi-Resolution Scheme is applied to reduce the virtual flight map grid number. An initial flight map is planned with moderate grid size. The initial flight map is refined by a more precise flight map (with smaller grid size) constructed on and near the initial flight path.

**Figure 7.**
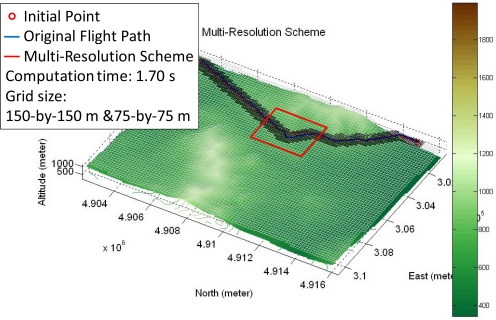

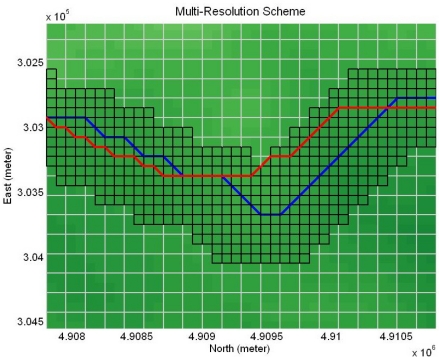
The flight path is planned by using the FPPS with the Multi-Resolution Scheme.

For the terrain used in this work, the computation time is more than 3 seconds with a 75-by-75 meters grid. However, it is less than 2 seconds if the Multi-Resolution Scheme is applied with 150-by-150 meters and 75-by-75 meters grids. The result is depicted in Figure 7.

### 3.3. Path Smoothing Method

**Figure 8.**
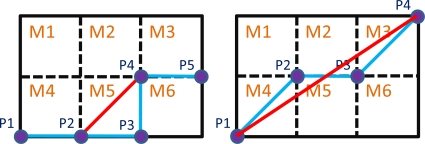
The example diagram of the path smoothing method.

In practice, there are many large turning angles in the virtual flight map, but the real flight track allows only limited turning angles and it is smoother than the planned flight path. Therefore, two path smoothing methods are used to restrain sudden turns. The concepts of the path smoothing methods are shown in Figure 8, where the blue line is the initial flight path and the points are the flight turn points. The first path smoothing method (left sub-figure) examines the two continuous line segments, $\bar{P2P3}$ and $\bar{P3P4}$. If the lines $\bar{P2P3}$. And $\bar{P3P4}$ are perpendicular to each other, the flight turn point *P*3 will be eliminated, and the new flight path $\bar{P2P4}$ (red line) substitutes for the old path $\bar{P2P3P4}$ (blue line.) However, if the point *P*3 is higher in altitude than the other points in the grid *M*5, the flight path $\bar{P3P4}$ will not replace the path $\bar{P2P3P4}$.

**Figure 9.**
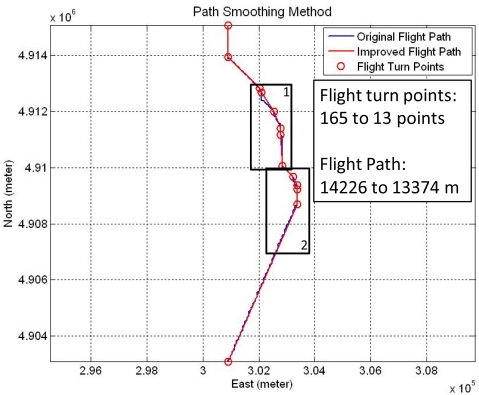

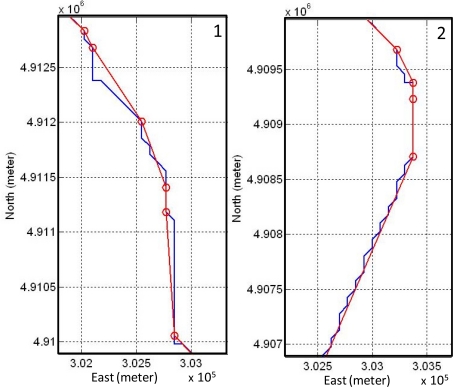
The flight turn points are decreased from 169 to 13 points and the route is shortened from 14.6 km to 13.7 km.

As shown in the right sub-figure in Figure 8, another path smoothing method is applied to reduce the flight turn points. The method calculates the tendency from the initial point (*P*1) to the last flight turn point (*P*3) and the next line segment (*i.e.*, $\bar{P2P3}$). If the included angle of the tendency and the last line segment is smaller than the threshold of the included angle, the next flight turn point (*P*4) is taken as the last one and checked again until all the included angles are larger than the threshold. The line $\bar{P1P4}$ (red line) is the new flight path that reduces the redundant flight turn points, *P*2 and *P*3. Figure 9 is the result by using the path smoothing methods, where the blue line is the initial flight path, the red line is the improved flight path, and the circles are the new flight turn points. The bottom sub-figures show the zoom-in views which are the black rectangles in the upper one. The flight turn points are decreased from 169 to 13 points, and the route is shortened from 14.6 km to 13.7 km. The original one has some sharp turn points because the virtual flight map is constructed by squares. After applying the Path Smoothing Method, the new flight path is smoother. In this section, the FPPS flight path planning capability is demonstrated by simulations, and the three methods used to improve the FPPS are also shown. With the planned flight path, the unmanned helicopter can avoid hazardous areas using the forbidden zone. The Multi-Resolution Scheme is used to reduce the computation time, and the path smoothing method improves the quality of the flight path planned by the FPPS.

## 4. Flight Control System

**Figure 10.**
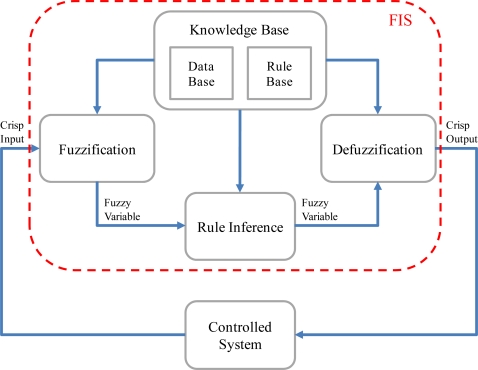
The architecture of the FIS includes three parts: the Fuzzification, the Rule Inference, and the Defuzzification.

When the flight path is laid out, a flight control system (FCS) is required so that the unmanned helicopter can follow the planned flight path and execute the mission. In this paper, the autonomous flight control of the unmanned helicopter is implemented by using the fuzzy logic control. The fuzzy logic control includes the throttle controller, the longitudinal controller, the lateral controller, and the tail controller. The fuzzy logic controller is designed using operational experience and expert knowledge, without knowing the actual dynamic model [13]. The FIS consists of three parts: the Fuzzification, the Rule Inference, and the Defuzzification. Figure 10 shows the architecture of the fuzzy inference system (FIS). In the FIS, the Rule Inference masters the input fuzzy variables into the output fuzzy variables according to the IF-THEN rules in the Knowledge Base. Tables 1 and 2 respectively depict the single input single output (SISO) and the multi input single output (MISO) Rule Inferences. ZE (zero) together with the combination of the letters P (positive), N (negative), L (large), M (medium) and S (small) describe symbolically the values in Tables 1 and 2. They are replaced by numerical values in practical applications. There are generally four flight controllers for a helicopter, namely the cyclic, the collective, the anti-torque pedal, and the throttle controllers [18]. The cyclic controller cyclically changes the pitch of the rotor blades to alter the longitudinal motion and the lateral motion of the helicopter in the horizontal plane. At the same time, the collective controller changes the pitch angles of all the main rotor blades. If a correction of the collective controller is made, the blades change accordingly, and thus the helicopter increases or decreases altitude. The antitorque pedal is similar to the rudder pedal in a fixed-wing aircraft. It changes the pitch angle of tail rotor and controls the helicopter heading. The throttle controller controls the engine power output, and it is connected to the rotor by a transmission. The function of the throttle controller is to maintain adequate engine power output to generate enough lift for flight.

**Figure 11.**
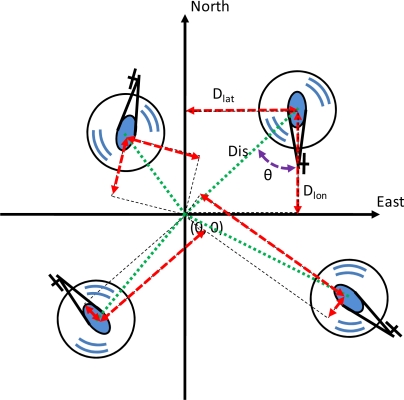
The concept diagram of (7). *Dis* is transformed into *D_lon_* and *D_lat_* according to this equation.

In order to control the unmanned helicopter and take advantage of the hovering capability, the FCS provides two operation modes, the hovering mode and the guidance mode. The hovering mode is composed of four FISs, and the guidance mode has five. The FISs for the hovering mode are shown in Equation (4):

(4) $$\begin{array}{l} \mathrm{Hovering}\;\mathrm{Mode}\\ C_{T}=\mathrm{FIS}\left(\mathrm{Altitude}\right)\\ C_{E}=\mathrm{FIS}\left(\mathrm{Pitch},\;D_{\mathrm{lon}}\right)\\ C_{A}=\mathrm{FIS}(\mathrm{Roll},\;D_{\mathrm{lat}})\\ C_{R}=\mathrm{FIS}\left(\mathrm{Heading}\right) \end{array}$$

where the throttle controller is a single-input-single-output (SISO) system which uses elevation error as the input data to infer the output data of the throttle setting (*C_T_*). Although the collective controller can also be used to control the throttle, it is closely related to the throttle controller, and it is left out because the throttle controller is more intuitive [19]. The cyclic controller is divided into the longitudinal controller and the lateral controller. For the longitudinal controller, the pitch angle error and the difference between the hovering center and the longitudinal direction of the unmanned helicopter (*D_lon_*) are used as the input variables, and the output data is the elevator correction (*C_E_*) which makes the unmanned helicopter tilt forward or backward. For the lateral controller, the roll angle error and the difference between the hovering center and the lateral direction of the unmanned helicopter (*D_lat_*) are used for the input variables, and the output data is the aileron correction (*C_A_*) which makes the unmanned helicopter move in the side direction. The variables *D_lon_* and *D_lat_* are defined in Equation (5):

(5) $$\begin{array}{l} D_{\mathrm{lon}}=-\mathrm{Dis}\times \text{cos}\left(\theta \right)\\ D_{\mathrm{lat}}=\mathrm{Dis}\times \text{sin}\left(\theta \right) \end{array}$$

where *Dis* is the distance between the hovering center and the position of the unmanned helicopter, and *θ* is the include angle of the hovering center and the unmanned helicopter. The concept of Equation (5) is illustrated in Figure 11, and *Dis* is transformed into *D_lon_* and *D_lat_* according to this equation. The tail controller is the anti-torque pedal controller that uses the heading angle error to infer the rudder correction (*C_R_*). By switching to the hovering mode, the FCS is able to maintain the attitude of the unmanned helicopter as well as hovering in space. The FISs for the guidance mode are depicted in Equation (6):

(6) $$\begin{array}{l} \mathrm{Guidance}\;\mathrm{Mode}\\ C_{T}=\mathrm{FIS}\left(\mathrm{Altitude}\right)\\ C_{E}=\mathrm{FIS}\left(\mathrm{Pitch},\;\;\mathrm{Dis}\right)\\ C_{A}=\{\begin{array}{c} \mathrm{FIS}\left(\mathrm{Roll},\;\;\psi \right)\\ \mathrm{FIS}\left(\mathrm{Roll},\;\;D_{\mathrm{lat}}\right) \end{array}\\ C_{R}=\mathrm{FIS}\left(\mathrm{Heading}\right) \end{array}$$

where the throttle controller and the tail controller are those in hovering mode. The longitudinal controller uses the pitch angle error and the distance between the next flight turn point and the unmanned helicopter, *Dis*, as the input variables. Therefore, when the distance is far from the current position, the unmanned helicopter will tilt the cyclic angle forward to fly to the next flight turn point or the destination. A limitation of the cyclic angle is set to prevent the controller from rectifying too much. The lateral controller includes two FISs, and it determines the aileron correction (*C_A_*) according to the FIS that has the maximum input error.

**Figure 12.**
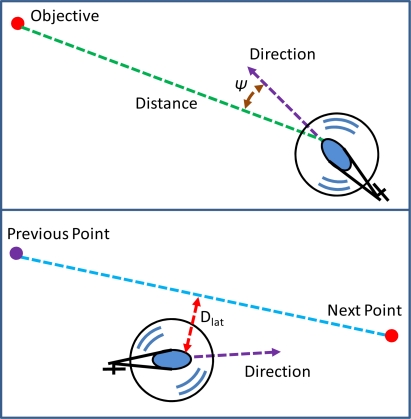
The concept diagram of the lateral controller of the guidance mode.

The first FIS uses the roll angle error and *ψ* to be the input variables, and *ψ* is the angle difference between the required heading angle and the nose direction angle [18,19]. The second FIS uses the roll angle error and *D_lat_* to calculate the output data, and *D_lat_* is the lateral distance error between the unmanned helicopter and the planned flight path. Figure 12 shows the concept of the lateral controller, where the top sub-figure is the first FIS, and the bottom sub-figure is the second FIS. In summary, the constructed FCS satisfies the requirements of the designated task, and it controls the unmanned helicopter to maneuver and hover in space. The architecture of the integrated system, and the associated experiments and the simulation results will be presented in the next section.

## 5. Simulation and Experiment Results

In this section, an integrated system to combine the FPPS and the FCS is proposed. The integrated system is implemented by using the flight simulation software, X-Plane. In Section 5.1, the unmanned helicopter is operated to hover in the space to examine the system performance. In Section 5.2, two DEMs on the complex terrain are used to simulate different environments in order to verify the performance of the integrated system. The unmanned helicopter’s real time position and attitude will be shown on the MATLAB indicators to monitor the vehicle’s states.

### 5.1. Flight Control System Test

**Figure 13.**
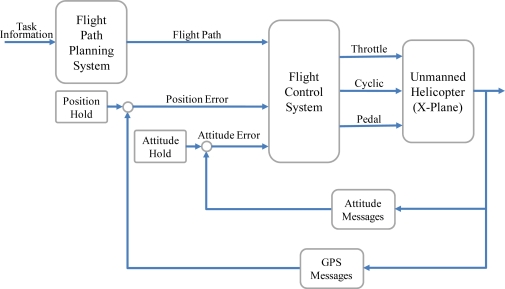
The architecture of the integrated system.

Two software tools are utilized to simulate the flight condition for the integrated system in this paper. The computing software, MATLAB, is used to construct the integrated system, and the flight simulation software X-Plane is used to perform the experiments. MATLAB and X-Plane are connected by the User Datagram Protocol (UDP) which is a core member of the Internet Protocol Suite [20]. The architecture of the integrated system is presented in Figure 13. When the user defines the starting and destination points, the FPPS maps out a flight path and delivers it to the FCS. After the FCS receives the attitude and GPS messages from X-Plane through the UDP, the system sends the output correction with the Knowledge Base through the UDP too. The X-Plane then corrects the control deflections and displays the simulation result via a three dimensional animation. The unmanned helicopter used in the experiment is the Raptor 30 V2 RC helicopter. The Raptor 30 V2 is a highly maneuverable helicopter that weights 3 kg. The fuselage length and width of the Raptor 30 V2 are 1,150 mm and 140 mm respectively. In addition, MATLAB and X-Plane are operated on a single computer, and the same local IP address, (127.0.0.1) is used as the remote IP address. Therefore, both software tools are synchronized on the same computer to avoid any message delays.

**Figure 14.**
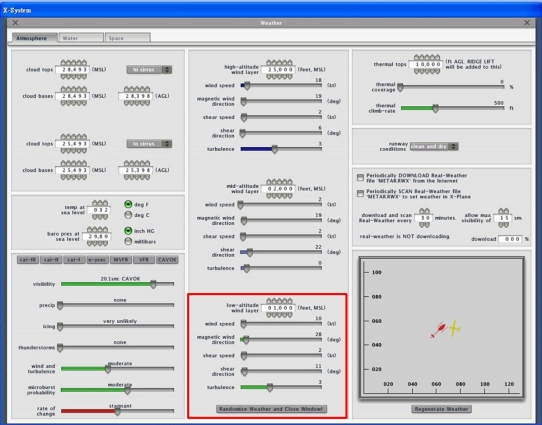
Stable weather settings.

In order to simulate real weather conditions, the Randomize Weather function of X-Plane is turned on. In the simulation, the system is test under the ideal weather without wind and randomness and a stable weather of 10 knots (4.5 m/s) wind speed, 2 knots (0.9 m/s) shear speed, and level 3 turbulence. The details of the stable weather conditions are shown in Figure 14.

**Figure 15.**
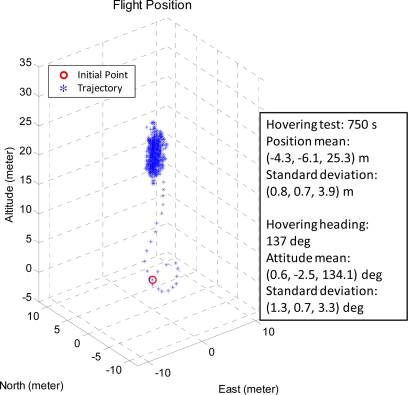

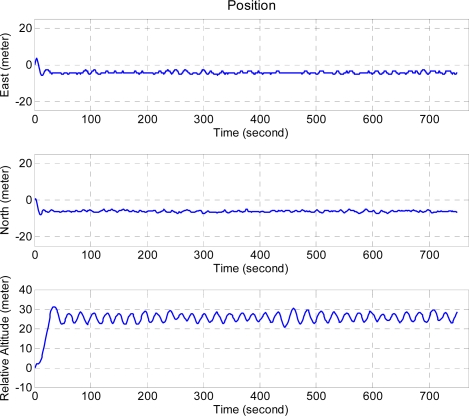
The hovering test result of the FCS under the ideal weather conditions.

**Figure 16.**
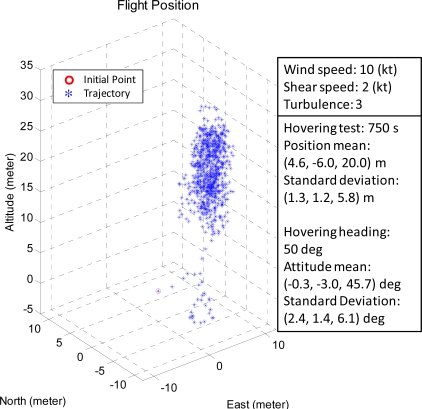

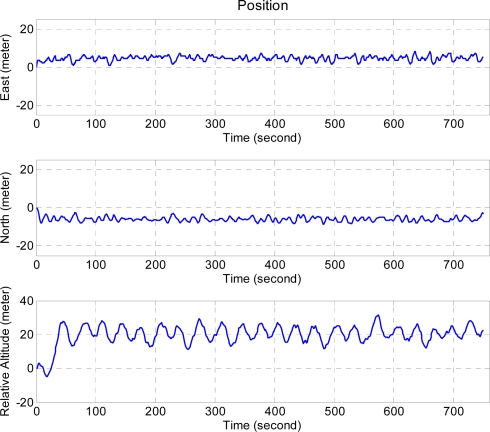
The hovering test result of the FCS under stable weather conditions.

Figures 15 and 16 are the unmanned helicopter trajectory under the ideal weather conditions and the stable weather conditions, respectively.

The mean and standard deviations of the positions are (−4.3, −6.1, 25.3) and (0.8, 0.7, 3.9) meters, respectively, for ideal weather while the corresponding values are (4.6, −6.0, 20.0) and (1.3, 1.2, 5.8) meters. The FCS is able to control the unmanned helicopter to hover on the specific area, and it is robust to operate in the real environment.

**Figure 17.**
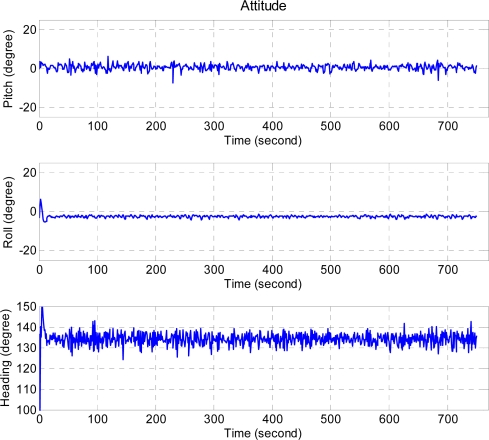
The performance of the attitude control under ideal weather condition, and the desired attitudes are (0, 0, 137.0) degrees which are pitch, roll, and heading angles, respectively.

Figure 17 presents the unmanned helicopter attitude under the ideal weather by setting the target attitude (pitch, roll, heading) as (0, 0, 137.0) degrees and the mean of the attitudes are (0.6, −2.5, 134.1) degrees and the standard deviations of the attitudes are (1.3, 0.7, 3.3) degrees.

**Figure 18.**
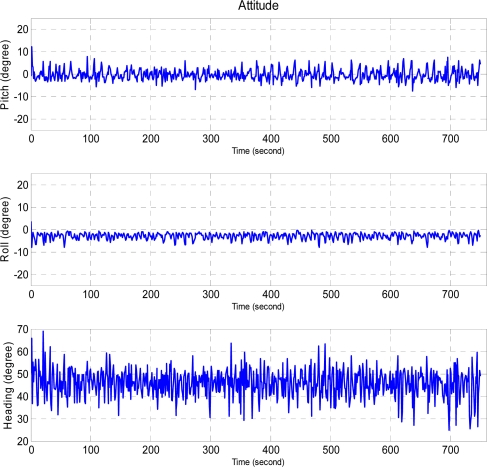
The performance of the attitude control under stable weather condition, and the desired attitudes are (0, 0, 50.0) degrees which are pitch, roll, and heading angles, respectively.

The corresponding values for the same unmanned helicopter targeted at (0, 0, 50.3) degrees are (−0.3, −3.0, 45.7) and (2.4, 1.4, 6.1) degrees respectively, as shown in Figure 18. The attitude result of the stable weather case is noisy, but FCS is capable of controlling the attitude of the unmanned helicopter under these conditions.

**Figure 19.**
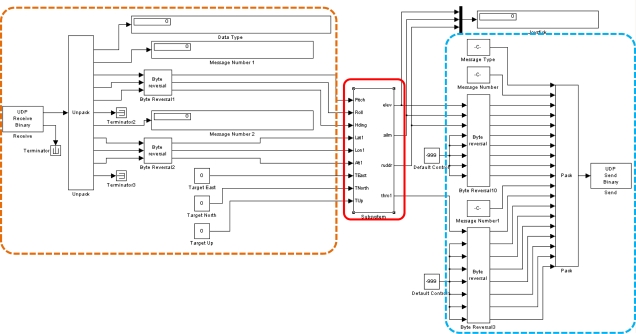
The architecture of the FCS in SIMULINK.

The FCS is implemented by using MATLAB/SIMULINK simulation program. The Rule Inferences are described in tables started from Table 3 to Table 6, and the Rule Inferences are developed using experience and testing rather than systematic identification or optimization. Figure 19 shows the architecture of the FCS in MATLAB/SIMULINK, where the orange and blue dotted line blocks are the UDP interfaces that connect with the X-Plane, and the red block is the FCS.

**Figure 20.**
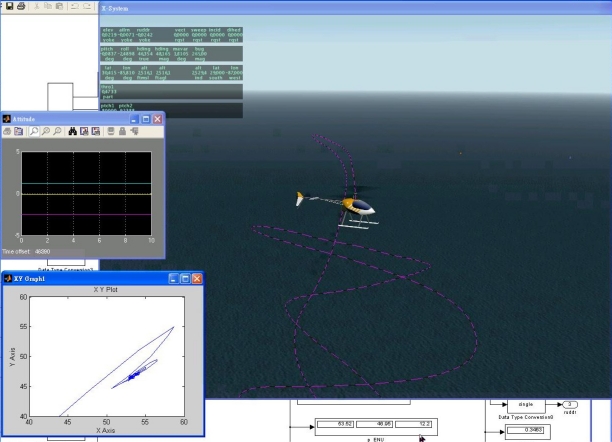
The FCS directs the unmanned helicopter to (50, 50, 30), where the main sub-figure is X-Plane, and two sub-figures on left are the FCS real time monitors to show the attitude and trajectory in the East-North horizontal plane.

Figure 20 illustrates the flight test simulation. The unmanned helicopter is directed to fly to a position at (50, 50, 30) meters relative to the initial point and hovers at the destination point. The main figure shows the X-Plane simulation in a three-dimensional animation, and the purple dashed line is the recorded trajectory of the unmanned helicopter. There are two sub-figures in Figure 20: the upper left plot shows the unmanned helicopter’s attitude and the lower left plot shows the trajectory in the East-North horizontal plane. Both of them are the real time indicators of the FCS.

### 5.2. Integrated System Test

**Figure 21.**
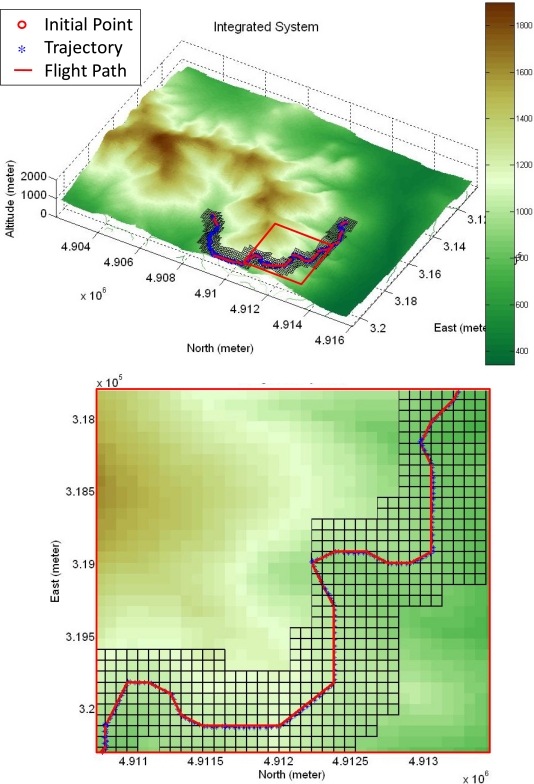
The test result of the integrated system.

**Figure 22.**
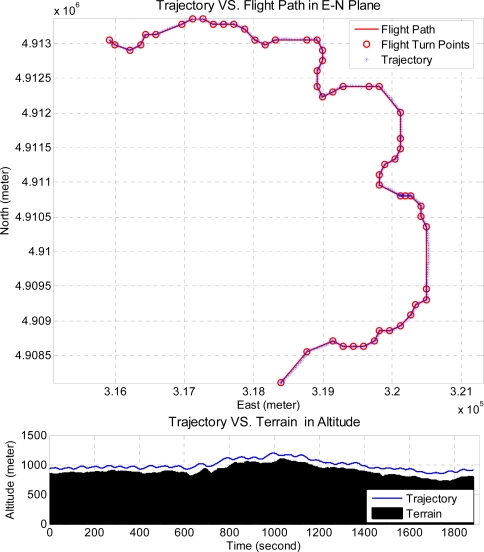
The upper sub-figure is the result in the East-North horizontal plane and the lower sub-figure is the altitude result.

In order to demonstrate the integrated system performance, the integrated system is commanded to pilot the unmanned helicopter to the destination through a mountainous and a valley terrain. The altitude of the mountainous terrain is more diverse than that of the valley terrain in which the turning angle along the horizontal plane of the valley terrain is generally larger. Figure 21 shows the test result of the integrated system with the “MtWashington-ft.grd” in MATLAB Mapping Toolbox as the mountainous terrain, and the bottom sub-figure shows the zoom-in views of the upper one. In Figure 22, the recorded trajectory and the planned flight path on the mountainous terrain are shown. The lower sub-figure shows the unmanned helicopter’s altitude, where the blue line is the actual unmanned helicopter’s trajectory, and the black colored portion is the terrain. One can observe from the figure that all the altitude differences between the recorded trajectory and the terrain are larger than the predefined vertical safety distance, and the flight trajectory closely follows the planned flight path.

**Figure 23.**
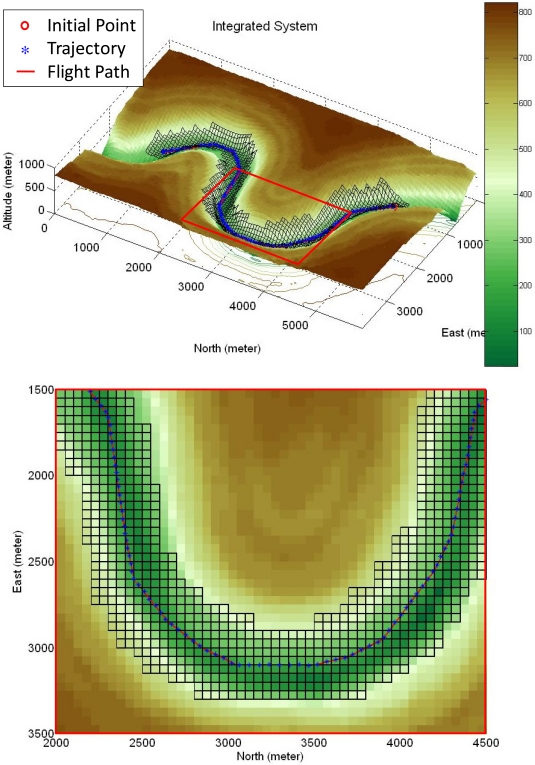
The test result of the integrated system.

**Figure 24.**
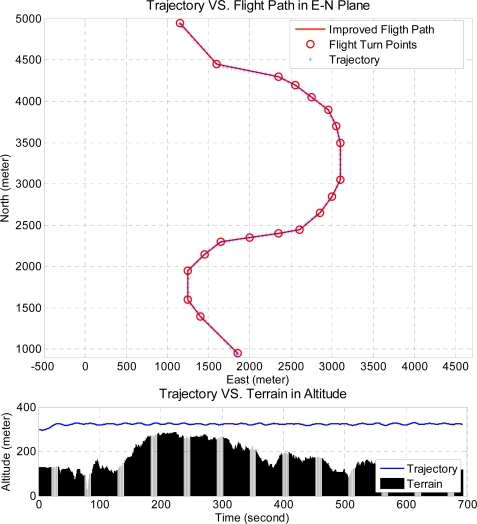
The resulting trajectory in the horizontal plane and the corresponding altitude result.

In addition, a virtual valley terrain is created and applied to the integrated system to evaluate the integrated system performance. The planned flight path is shown in Figure 23, where the large and small grid sizes are of 75-by-75 and 50-by-50 meters, respectively, and the cruising altitude is set at 300 meters. This figure also shows the result of the simulation, and the bottom sub-figure is a zoom-in view of the red square. In Figure 24, the upper sub-figure is the result in the East-North horizontal plane and the lower sub-figure is the altitude result. As shown in Figure 24, one can observe that the integrated system is capable of controlling the unmanned helicopter to follow the planned flight path smoothly and safely in the valley, and the performance of the integrated system is validated in these two difficult terrains.

According to the simulation results, the integrated system is able to generate an optimized flight path, and controls the unmanned helicopter to follow the planned flight path and reach the destination smoothly and safely.

## 6. Conclusions

An integrated system is presented in this paper to navigate and guide an unmanned helicopter. The integrated system consists of the flight path planning system (FPPS) to determine the proper flight path and the flight control system (FCS) to control the helicopter. The integrated system is implemented in MATLAB/SIMULINK and the X-Plane software to investigate its performance under different scenarios. As shown in the simulations and the experimental results, the integrated system is capable of generating flight paths for all kinds of digital elevation models (DEMs) and digital terrain models (DTMs). The flight path is planned by using the least-cost method to reduce the operation cost, and the integrated system can plan different flight paths for different unmanned helicopters according to their parameters. Moreover, the integrated system developed in this work is able to immediately generate a new flight path if an unknown obstacle is detected by an onboard sensor or if the assigned mission is changed. As shown in the simulation results, the integrated system is able to control the unmanned helicopter under different weather conditions, and the integrated system can safely navigate and guide the unmanned helicopter over different terrains.

## Figures and Tables

**Table 1.** Single Input Single Output (SISO) Rule Inference.

Variable	NL	NM	NS	ZE	PS	PM	PL
Control value	PL	PM	PS	ZE	NS	NM	NL

**Table 2.** Multi Input Single Output (MISO) Rule Inference.

**Variable 1**	**NL**	**NM**	**NS**	**ZE**	**PS**	**PM**	**PL**
**Variable 2**							
NL	PL	PL	PL	PL	PM	PS	ZE
NM	PL	PL	PL	PM	PS	ZE	NS
NS	PL	PL	PM	PS	ZE	NS	NM
ZE	PL	PM	PS	ZE	NS	NM	NL
PS	PM	PS	ZE	NS	NM	NL	NL
PM	PS	ZE	NS	NM	NL	NL	NL
Pl	ZE	NS	NM	NL	NL	NL	NL

**Table 3.** Pitch Rule Inference.

	**NL**	**NM**	**NS**	**ZE**	**PS**	**PM**	**PL**
Error (degree)	−20	−10	−2	0	2	10	20
Control (%)	−45	−30	−15	0	15	30	45

**Table 4.** Roll Rule Inference.

	**NL**	**NM**	**NS**	**ZE**	**PS**	**PM**	**PL**
Error (degree)	−20	−10	−2	0	2	10	20
Control (%)	−45	−30	−15	0	15	30	45

**Table 5.** Heading Rule Inference.

	**NL**	**NM**	**NS**	**ZE**	**PS**	**PM**	**PL**
Error (degree)	−8	−4	−2	0	8	16	30
Control (%)	9	17	21	25	35	60	80

**Table 6.** Throttle Rule Inference.

	**NL**	**NM**	**NS**	**ZE**	**PS**	**PM**	**PL**
Error (degree)	−8	−4	−2	0	8	16	30
Control (%)	9	17	21	25	35	60	80
